# Estimating the Population Size of Female Sex Workers in Namibia Using a Respondent-Driven Sampling Adjustment to the Reverse Tracking Method: A Novel Approach

**DOI:** 10.2196/11737

**Published:** 2019-03-14

**Authors:** Paul Douglas Wesson, Rajatashuvra Adhikary, Anna Jonas, Krysta Gerndt, Ali Mirzazadeh, Frieda Katuta, Andrew Maher, Karen Banda, Nicholus Mutenda, Willi McFarland, David Lowrance, Dimitri Prybylski, Sadhna Patel

**Affiliations:** 1 Center for AIDS Prevention Studies Division of Prevention Science University of California, San Francisco San Francisco, CA United States; 2 Strategic Information/Monitoring and Evaluation WHO India Country Office Gurugaon, Haryana India; 3 Directorate of Special Programmes Response Monitoring & Evaluation Subdivision Ministry of Health and Social Services Windhoek Namibia; 4 Division of Global HIV and Tuberculosis US Centers for Disease Control and Prevention Atlanta, GA United States; 5 Institute for Global Health Sciences University of California San Francisco San Francisco, CA United States; 6 Department of Epidemiology and Biostatistics University of California, San Francisco San Francisco, CA United States

**Keywords:** population density, social networking, sex workers, vulnerable populations, human immunodeficiency virus

## Abstract

**Background:**

Key populations, including female sex workers (FSWs), are at a disproportionately high risk for HIV infection. Estimates of the size of these populations serve as denominator data to inform HIV prevention and treatment programming and are necessary for the equitable allocation of limited public health resources.

**Objective:**

This study aimed to present the respondent-driven sampling (RDS) adjusted reverse tracking method (RTM; RadR), a novel population size estimation approach that combines venue mapping data with RDS data to estimate the population size, adjusted for double counting and nonattendance biases.

**Methods:**

We used data from a 2014 RDS survey of FSWs in Windhoek and Katima Mulilo, Namibia, to demonstrate the RadR method. Information from venue mapping and enumeration from the survey formative assessment phase were combined with survey-based venue-inquiry questions to estimate population size, adjusting for double counting, and FSWs who do not attend venues. RadR estimates were compared with the official population size estimates, published by the Namibian Ministry of Health and Social Services (MoHSS), and with the unadjusted RTM.

**Results:**

Using the RadR method, we estimated 1552 (95% simulation interval, SI, 1101-2387) FSWs in Windhoek and 453 (95% SI: 336-656) FSWs in Katima Mulilo. These estimates were slightly more conservative than the MoHSS estimates—Windhoek: 3000 (1800-3400); Katima Mulilo: 800 (380-2000)—though not statistically different. We also found 75 additional venues in Windhoek and 59 additional venues in Katima Mulilo identified by RDS participants’ responses that were not detected during the initial mapping exercise.

**Conclusions:**

The RadR estimates were comparable with official estimates from the MoHSS. The RadR method is easily integrated into RDS studies, producing plausible population size estimates, and can also validate and update key population maps for outreach and venue-based sampling.

## Introduction

### Background

Over the past decade, population size estimation (PSE) has become increasingly important in the global fight against the HIV epidemic, particularly among key populations [1,2]. Key populations—for example, men who have sex with men, female sex workers (FSWs), and people who inject drugs—face a disproportionate burden of HIV infection, relative to the general population, because of behavioral risk factors [3-5]. Size estimation for key populations is necessary to estimate the absolute burden of disease by serving as the denominator for the population at risk [6]. Furthermore, PSE for key populations is used to evaluate the reach and coverage of existing services for key populations as well as for program planning and allocation of limited public health resources to most effectively combat local HIV epidemics [7]. PSE of key populations is also necessary for tracking progress toward 90-90-90 diagnosis and treatment targets: by 2020, 90% of people living with HIV (PLWH) knowing their status, 90% of diagnosed PLWH receiving antiretroviral treatment, and 90% of PLWH on treatment being virally suppressed [8]. Without PSE, investigators and policy makers cannot translate these proportional targets into absolute numbers to ensure appropriate resource allocation. Currently, there is no gold standard method to estimate the size of key populations [9]. Instead, researchers must choose from a menu of different PSE methods, each leveraging information about the population size from various data sources while also being vulnerable to different biases [6].

The reverse tracking method (RTM), for example, is an underutilized PSE method that leverages information about the venues visited by a key population to estimate population size [10]. The RTM takes a 2-stage approach to size estimation. During the first stage, researchers map out the venues where the key population can be found (the venue-based sampling frame) and obtain the size for each venue, *M*_*i*_ (ie, how many members of the key population can be found there), typically from at least 3 key informants at each venue or from direct observation by the research team. During the second stage, the researchers return to either all or a sample of the venues from the sampling frame, sampled with probability proportional to the size of the venue, and directly count the number of people who are members of the key population for each venue, *N*_*i*_. The ratio of *N*_*i*_*/M*_*i*_, averaged over all the venues from the second visit, is a correction factor that is then multiplied by the sum of the counts from the first stage to estimate the population size.

Although the RTM creatively leverages venue-based data, the method relies on several strong assumptions. First, the RTM implicitly assumes that all members of the key population can be found at physical venues that are known to the researchers. Second, the method assumes that members of the key population exclusively *belong* to a single venue; the equation does not adjust for double counting [10]. In addition, to efficiently use resources allocated for research, this venue-based PSE method is best integrated into a study that uses venue-based sampling to find the key population, such as time location sampling (TLS) [11]. However, many surveillance studies of key populations use respondent-driven sampling (RDS), a peer referral–based sampling method [12]. As such, the RTM is inefficient, from a resource perspective, to include as a PSE method for RDS-based surveillance studies of key populations.

### Objectives

We sought to modify the RTM for the RDS study design. By including *venue inquiry* questions in the RDS survey to serve as a *virtual second visit* and combining that information with the size of mapped venues (ie, the number of members of the key population found at the mapped venue) obtained before the RDS survey during the formative assessment stage, we successfully developed a novel PSE method that leverages venue information within the RDS context. With additional information from the survey, we adjusted our size estimate for double counting (accounting for people who visit multiple venues) and for people who do not attend any venues, thereby overcoming notable limitations of the original RTM. We refer to this approach as the RDS-adjusted RTM (RadR, pronounced “radar”). In this paper, we describe the RadR method and demonstrate its implementation using FSW data from an integrated biobehavioral surveillance study (IBBSS) in Namibia as a case example.

## Methods

### Study Design

From September 2012 to June 2014, the Namibian Ministry of Health and Social Services (MoHSS) partnered with the US Centers for Disease Control and Prevention (CDC) and the University of California, San Francisco (UCSF), to conduct a cross-sectional IBBSS of FSWs. The purpose of this study was to measure the prevalence of HIV infection among the FSW population, to assess HIV-related risk, preventive, and health-seeking behaviors, and to estimate the size of the FSW population in selected urban areas. The IBBSS was implemented in 4 sites on the basis of HIV prevalence from sentinel surveillance and the availability of community-based organizations and other organizations providing services to the FSW population. The IBBSS took place in Windhoek (the capital and largest city), Walvis Bay and Swakopmund (neighboring cities on the coast), Oshikango and Oshikati (neighboring towns in the northern region), and Katima Mulilo (a border town in the northeastern region, receiving traffic from Angola, Botswana, Zambia, and Zimbabwe) [13].

### Study Subjects

Participants were recruited for the IBBSS using RDS. RDS is a social networking–based sampling and analytic approach [14,15]. Briefly, the sampling process begins with a selection of *seeds* members of the target population purposively selected by the research team, to often represent the presumed diversity of the target population. Each seed is given a limited number of coupons (eg, 3), to recruit other members of the target population (eg, FSWs) from within their social network to participate in the study. After participating in this study, recruiters are also given a limited number of coupons to recruit people from their social network. These coupons are used to participate in the study and for the researchers to track who was recruited by whom. In addition to the primary incentive that everyone receives for participating in the study, recruiters also receive a secondary incentive for every one of their recruits who successfully participates in the study. This process of recruitment continues until the sample size is reached and equilibrium (ie, when additional recruitment does not substantially change the sample characteristics/proportions) is achieved for selected variables. In theory, with enough waves of recruitment and adjustment for the network recruitment design, the final RDS sample will approximate a representative sample of the target population, independent of the characteristics of the initial seeds. The number and selection of seeds are also guided by practical study implementation considerations (eg, people well connected to the social network to begin and facilitate active recruitment and chosen for diversity with respect to key demographic and behavioral variables). The MoHSS report provides the results of the main objectives of the IBBSS as well as specifics on the implementation of the survey among FSWs [13]. For a more detailed discussion of RDS implementation, we refer the reader to Johnston et al’s systematic review of published RDS studies [12] and Gile et al’s study on RDS diagnostics [16].

For the Namibia IBBSS, participants were eligible for the study if they met the following criteria: were at least 18 years of age, biologically female, able to speak English, Oshiwambo, Silozi, or Afrikaans, exchanged vaginal, anal, or oral sex for money during the 30 days preceding the IBBSS, and were residents of the study area for at least 6 months preceding the IBBSS. In total, 12 seeds were selected in Windhoek and 8 seeds were selected in Katima Mulilo to represent the diversity of the population with respect to age, cultural and linguistic background, and socioeconomic status. Recruitment was conducted from September 2012 to August 2013 for Windhoek and from October 2013 to June 2014 for Katima Mulilo. The number of coupons distributed to each person ranged from 3 to 11 to facilitate a diverse sample and aid the progress of recruitment in each city.

For the purpose of demonstrating the RadR method, we restricted our analysis to 2 of the 4 IBBSS sites, Windhoek and Katima Mulilo. Unlike the other sites, the Windhoek and Katima Mulilo sites did not combine multiple towns into a single site.

The mapping and count, the first stage of the RTM, took place during a 3-week formative assessment period beginning 3 weeks before the start of the RDS survey. Venues where FSWs congregate and find clients were identified on the basis of key informant interviews and focus group discussion with members of the FSW community who were identified by local nongovernmental organizations (NGOs) providing services to key populations in Namibia. Key informants were asked to name and identify known or frequented venues or hotspots. A list and map of venues were compiled for each population in each site. As fewer than 30 venues were named in each site, a complete census was taken at all venues identified. Types of venues identified by key informants principally included bars, night clubs, hotels and guesthouses, streets, as well as petrol and service stations. Study teams and individuals familiar with the local context visited the venues for observation and direct counts of the study populations for approximately 5 hours on the peak times indicated by key informants.

### Measures

As a *virtual second visit*, we asked each RDS participant to name the venues that she goes to most frequently to find or solicit clients. Specifically, participants were asked, “What is the name of the venue you go to most frequently to find clients?” and “In the past 30 days, how often did you attend this venue?” Participants could list up to 3 venues or respond that they do not go to venues to find clients. Responses were open-ended; participants provided names of the venues they attended most frequently as opposed to selecting from a predetermined list. After data collection, all responses were tabulated and summed by venue.

### Data Analysis

**Figure 1:**
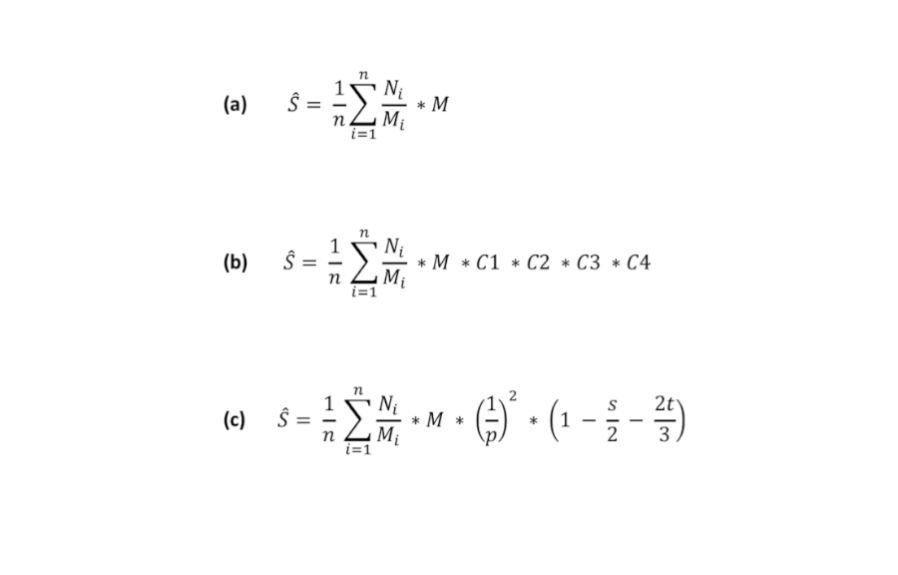
Equations: (a) Reverse Tracking Method, (b) Respondent-driven sampling adjusted Reverse Tracking Method, and (c) simplified Respondent-driven sampling adjusted Reverse Tracking Method.

The RTM approach to size estimation is calculated according to equation (a) in Figure 1.

*Ŝ* is the estimated population size, *n* is the number of venues visited on the second visit, *N*_*i*_ is the number of people observed at venue *i* on the second visit, *M*_*i*_ is the number of people observed at venue *i* on the first visit (or reported by a key informant for venue *i*), and *M* is the total number of people observed at all venues on the first visit.

For the RadR method, *M* is the total number of people observed at all venues on the first visit (from the mapping and enumeration exercise during the formative assessment), *M*_*i*_ is the number of people observed at venue *i* during the first visit, and *N*_*i*_ is the number of people observed at venue *i* during the *virtual second visit*. The original RTM equation is insufficient for RDS studies as the virtual second visit (*N*_*i*_) is taken from the RDS sample that reports attending venue *i* instead of from the target population that is physically observed at venue *i*. We therefore augmented the original RTM equation with additional correction factors to standardize to the target population and leverage additional information provided by the RDS survey through inverse probability weights. The augmented equation is given as equation (b) in Figure 1.

C1 (correction factor 1) is the *standardization parameter*, used to standardize the study population to the target population. It is calculated as 1/(R*/RDS), where R* is the number of RDS respondents who visited a mapped venue (ie, a venue included in the mapping phase), and *RDS* is the RDS sample size. The standardization parameter assumes that the RDS sample is representative of the target population. Formally, we assume the following equivalency, n/N=R*/RDS, where *n* is the number of people from the target population who are observed at venues, and *N* is the number of people in the target population. C2 (correction factor 2), the *visibility parameter*, accounts for the visible, or reachable, population; *M* is upweighted to account for people who attend venues previously unmapped, and they were therefore not a part of the original sampling frame. This parameter is calculated as (1−r)/p, where *r* is the proportion of the RDS sample that reports not going to any venues and *p* is the proportion of the RDS sample that reports attending a mapped venue. C3 (correction factor 3), the *hidden population parameter*, accounts for people who do not go to venues. This parameter is calculated as 1/(1−r). Finally, C4 (correction factor 4), the *double counting parameter*, accounts for people who go to multiple venues. This parameter is calculated as 1−(s/2)−(2t/3), where *s* is the proportion of venue-attending RDS participants who attend 2 venues, and *t* is the proportion of venue-attending RDS participants who attend 3 venues. For C1, R*/RDS is equivalent to *p* in C2. The equation for RadR then simplifies to equation (c) in Figure 1.

**Figure 2:**
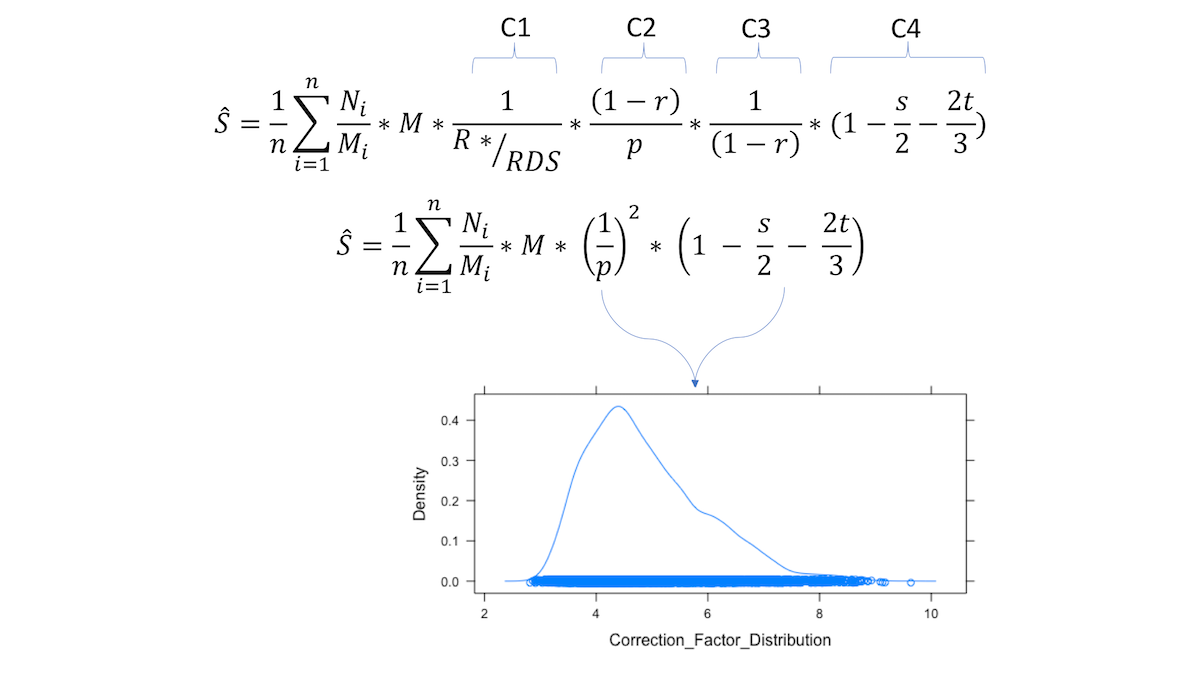
Respondent-driven sampling adjusted reverse tracking method equation (complete and simplified). Correction factors are calculated from the simulated distributions, from which the 2.5 percentile and 97.5 percentile are used to calculate 95% simulation intervals. Key: Ŝ=the estimated population size; n=the number of venues visited on the second visit; Ni=the number of people observed at venue i on the second visit; Mi=the number of people observed at venue i on the first visit; M=the total number of people observed at all venues on the first visit; R*=the sum of the number of times that a mapped venue is reported from the venue inquiry questions; RDS=the RDS sample size; r=the proportion of the RDS sample that reports not going to any venues; p=the proportion of the RDS sample that report attending a mapped venue; s=the proportion of venue-attending RDS participants who attend two venues; t=the proportion of venue-attending RDS participants who attend three venues.

To calculate 95% simulation intervals (SIs), we created probability distributions for the correction factors and resampled from those distributions. Drawing on the simplified RadR equation above, we fit the RDS-weighted values of *p*, *s*, and *t* (point estimates and 95% CI) to beta distributions. We assume a beta distribution here as this family of distributions is flexible and convenient for fitting quantities that are constrained to values between 0 and 1 [17]. Resampling from these distributions 10,000 times, we calculated the values of the simplified correction factors, storing the product, (1/p)^2^ * (1- (s/2) - (2t/3)), for each iteration, thus creating a distribution of the simplified correction factors. The 2.5 and 97.5 percentile values were then obtained and multiplied by the fixed values portion of the RadR equation to calculate the 95% SI. The calculations are illustrated in Figure 2.

To assess the performance of RadR method, we chose 2 comparisons. First, as RadR shares underlying assumptions and theoretically and practically builds upon the RTM method, we compare its results with the (unadjusted) results of RTM [6,10]. Second, to assess its acceptability and usefulness to policy makers, we also compared the results with the official PSE for FSWs from the MoHSS [13]. These results were adopted following a stakeholder consensus following a *modified Delphi* method [5,6,13,18]. Representatives from the MoHSS, CDC, the US Agency for International Development, local NGOs working with the FSW population, and FSW population members convened at a stakeholder workshop following data collection for the IBBSS. Each stakeholder provided an initial estimate for the FSW population in the study site on the basis of their experience with the population. These estimates were then allowed to be revised after stakeholders had the chance to discuss the rationale behind their estimates and after seeing the empirical results from several PSE methods that were included in the IBBSS (ie, key informant interview, unique object multiplier, wisdom of the crowd, and literature review, but not the RTM or RadR, which was not available at the time of the stakeholder meeting). The median of the revised stakeholder estimates was presented as the official population size estimate in the MoHSS report [13].

RDS-weighted values were calculated using RDS-A software version 0.42 (Handcock, Fellows, and Gile) [19]. The RDS-II estimator was used to calculate RDS-weighted point estimates and 95% CI [20]. Imputed visibility, a measure of a person’s connectedness in the social network, was used in place of network size for RDS-weighted estimates [21]. Self-reported network size may be a biased representation of a person’s position and influence in a network because of recall bias, digit preference for round numbers (eg, people reporting a network size of 20 rather than 23), and access to yet unsampled members of the target population at the time of recruitment. Imputed visibility overcomes these potential biases by leveraging self-reported network size, the time during the sampling process during which study participants were recruited, and the number of people study participants were able to recruit. R statistical software version 3.4.1 (R Core Team) was used to estimate 95% SIs [22].

### Ethics Approval

The protocol for the main IBBSS received approval from the Research Committee of the Directorate for Policy, Planning and Human Resources of the MoHSS in Windhoek, Namibia, the Committee on Human Research at the UCSF in San Francisco, California, USA, and the Division of Global HIV and Tuberculosis in the CDC, Atlanta, Georgia, USA. All study participants provided verbal informed consent before enrollment in the survey.

## Results

### Sampling/Recruitment

In Windhoek, 10 seeds were initially selected to begin recruitment and 9 additional seeds were added to increase the pace of recruitment. All 19 seeds were productive recruits, resulting in a total of 316 participants sampled over 7 waves of recruitment. Of the total number of coupons distributed in Windhoek, 28.8% (366/1271) were returned by potential participants. In Katima Mulilo, 9 seeds were identified to recruit for the study; however, 1 seed was found to be ineligible. The remaining 8 seeds were productive recruits, resulting in 309 participants sampled over 11 waves of recruitment. Of the total number of coupons distributed in Katima Mulilo, 48.0% (426/887) were returned by potential participants. FSWs were younger in Katima Mulilo (mean age 27.3 years) compared with Windhoek (mean age 30.3 years). The majority of FSWs in both locations had achieved at least a secondary school education. FSWs in Windhoek reported more client partners in the 30 days preceding the interview compared with FSWs in Katima Mulilo. RDS-weighting of the sample indicated that over half of the FSWs in Katima Mulilo are HIV positive (56.8%; 177/309) compared with nearly one-third of FSWs in Windhoek (32.6%; 103/316; Table 1).

**Figure 3:**
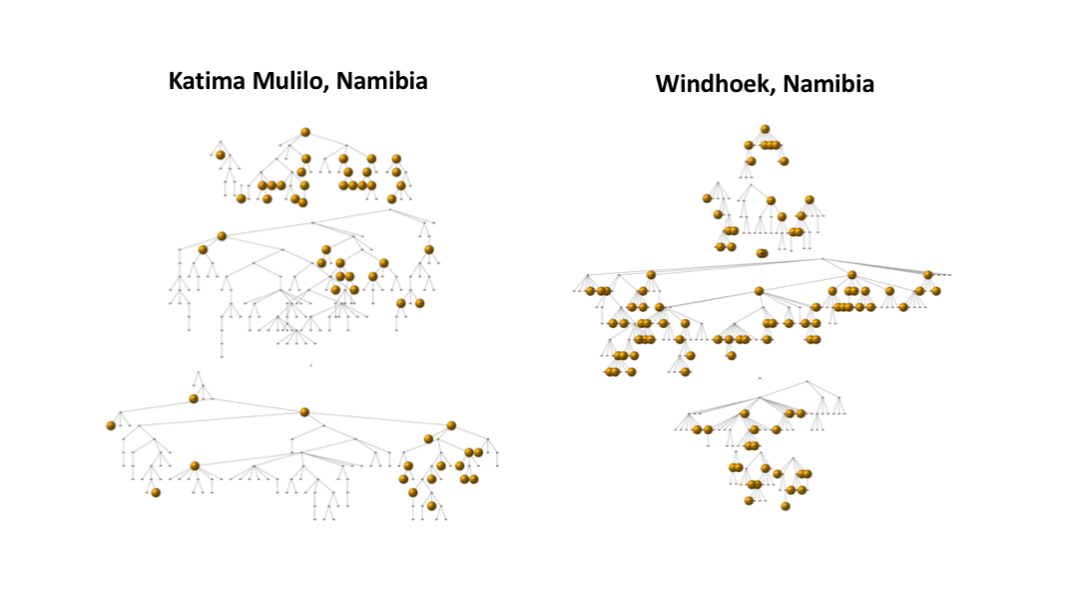
Respondent-driven sampling recruitment tree of female sex workers in Katima Mulilo, Namibia and Windhoek, Namibia. Large nodes indicate participants who report not attending venues to find clients.

In Windhoek, 31.6% (100/316) of FSWs reported not visiting venues to find clients; 37.6% (122/316) of FSWs also reported that they had visited at least one of the venues previously mapped by the research team during the formative assessment. In comparison, in Katima Mulilo, 17.0% (56/309) of FSWs reported not visiting venues to find clients; 42.3% (127/309) of FSWs also reported that they had visited at least one of the venues previously mapped by the research team during the formative assessment. This percentage increases to nearly 70% if venues are included that were mapped by the research team but no FSWs were observed by the research team during the enumeration exercise. Figure 3 depicts the RDS recruitment tree by site. Each node, representing a participant, is scaled according to venue attendance; large nodes indicate the participant did not visit venues to find clients. The venue inquiry questions identified over 75 previously unmapped venues in Windhoek and 59 previously unmapped venues in Katima Mulilo.

### Population Size

Using the RadR method, the FSW population size was estimated at 1552 (95% SI: 1101-2387) in Windhoek, corresponding to roughly 1.8% of the adult female population. The FSW population size in Katima Mulilo was estimated at 453 (95% SI: 336-656), corresponding to roughly 4.9% of the adult female population. Table 2 compares these estimates with the stakeholder consensus and the unadjusted RTM estimate.

**Table 1:** Demographics and descriptive statistics for respondent-driven sampling sample of female sex workers in Katima Mulilo and Windhoek, Namibia (because of rounding, percentages may not sum to 100%).

RDS^a^ participant characteristics		Katima Mulilo		Windhoek	
		n	RDS Adjusted % (95% CI)	n	RDS Adjusted % (95% CI)
Age (years), mean (minimum-maximum)		309	27.3 (18-53)	316	30.3 (18-65)
**Education**			
	Primary/less than primary	89	28.6 (22.9-34.1)	116	36.7 (30.6-42.7)
	Secondary	219	71.1 (70.4-71.7)	198	62.6 (61.9-63.3)
	Vocational/Technical	0	0	2	0.7 (0.0-6.8)
**Client partners during 30 days preceding interview**			
	<5	186	60.9 (55.1-66.9)	139	42.6 (36.8-48.4)
	5-9	88	27.9 (22.9-32.8)	94	30.9 (25.2-36.5)
	10-14	19	6.4 (3.6-9.2)	24	7.4 (4.3-10.4)
	>15	16	4.8 (2.1-7.4)	59	19.2 (15.0-23.3)
**Marital status**			
	Never married	250	81.6 (77.2-86.1)	276	87.8 (83.5-92.1)
	Previously or currently married	59	18.4 (14.0-22.8)	40	12.2 (7.9-16.5)
**HIV status§^b^**			
	Positive	177	56.8 (50.1-63.4)	103	32.6 (26.7-38.5)
	Negative	131	43.0 (36.3-49.6)	206	65.7 (59.7-71.7)
**Venues visited to find clients**			
	I do not go to venues	56	17.0 (11.5-22.4)	100	31.6 (26.1-37.2)
	1 venue	32	11.1 (7.2-15.0)	84	27.9 (22.5-33.4)
	2 venues	106	35.0 (28.7-40.3)	80	24.8 (19.7-30.0)
	3 venues	115	37.4 (31.8-43.1)	51	15.2 (11.0-20.0)
	Refuse to Answer		—^c^	1	—^c^
**Visited >1 of the mapped venues**			
	Yes	127	42.3 (35.1-49.5)	122	37.6 (30.2-44.9)
	No	182	57.7 (50.5-64.9)	194	62.4 (55.1-69.8)
**Correction factors**			
	C1: standardization parameter		2.36^d^		2.66
	C2: visibility parameter		1.96		1.82
	C3: hidden population parameter		1.21		1.46
	C4: double counting parameter		0.49		0.67

^a^RDS: respondent-driven sampling.

^b^§:Stratified counts do not sum to N because of indeterminate HIV test results.

^c^Not applicable.

^d^95% CI values are not applicable.

**Table 2:** Population size estimates of female sex workers by study site and population size estimation method (we use “Acceptable bounds” here as an umbrella term as some methods report 95% CIs, other methods report plausibility bounds, and the respondent-driven sampling adjusted reverse tracking method reports 95% simulation intervals).

Study site and population size estimates method		Estimated number of FSWs^a^ (acceptable bounds)	Estimated percentage of adult (15 to 49 years) female population who are FSWs (acceptable bounds)
**Windhoek**			
	Stakeholder consensus^b^	3000 (1800-3400)	2.2 (2.0-3.6)
	Reverse tracking method	492 (418-565)	0.56 (0.48-0.64)
	RadR^c^	1552 (1101-2387)	1.77 (1.25-2.72)
**Katima Mulilo**			
	Stakeholder consensus	800 (380-2,000)	8.6 (4.1-21.5)
	Reverse tracking method	192 (181-203)	2.06 (1.95-2.18)
	RadR	453 (336-656)	4.85 (3.60-7.03)

^a^FSWs: female sex workers.

^b^The stakeholder consensus was informed by the following population size estimates methods: key informant interview, unique object multiplier, wisdom of the crowd, and literature review.

^c^RadR: respondent-driven sampling adjusted reverse tracking method.

## Discussion

### Principal Results

We estimated the size of the FSW population to be 1552 (95% SI: 1101-2387) in Windhoek and 453 (95% SI: 336-656) in Katima Mulilo, using the RadR method. FSW size estimates were notably larger when using the RadR method compared with the unadjusted RTM (1552 vs 492 in Windhoek and 453 vs 192 in Katima Mulilo). This is expected as the RadR method was conceived to explicitly account for the *hidden* members of the key population in its calculation (ie, those who cannot be found at physical venues but participated in the RDS survey), whereas the unadjusted RTM relies only on the observable population when estimating the population size. Accounting for the *hidden* members is especially important in research involving key populations as the social marginalization often faced by these groups may result in a sampling bias that may be particularly strong for venue-based study designs. For example, we estimated that over 30% of the FSW population in Windhoek and 17% of the FSW population in Katima Mulilo would not be found at venues (Table 1, Figure 3). Therefore, we believe that incorporating such correction factors as the *hidden population parameter* improves the validity of the RadR population size estimates compared with the unadjusted RTM estimates.

The RadR estimates are consistent with results from the official FSW size estimates from the stakeholder consensus with respect to having overlapping CIs or SIs. Of note, in Katima Mulilo, there is a substantial difference between the stakeholder consensus estimate and the unadjusted RTM estimate. However, the RadR estimate is in closer agreement with the final stakeholder estimate, illustrating the impact of our correction factors on calculating a more plausible population size estimate. Still, the RadR estimate is slightly more conservative than the stakeholder consensus. This may be because of FSW demographic groups, such as higher income FSWs, who may not be observed in either the census mapping or the RDS study. This stratum of FSWs would then be absent from the RadR calculation, whereas stakeholders who have knowledge of this group may incorporate them into their estimation of the population size.

### Strengths

In addition to calculating plausible size estimates for the target population, the RadR method advances the PSE field and key population surveillance in 4 ways. First, the venue inquiry questions serve to validate the existing sampling frame and census mapping. Responses to these questions indicate whether the target population actually attends the venues identified during the formative assessment. In addition, the frequency with which a venue is reported in the survey provides some insight into the popularity of that venue among the target population, assuming that RDS recruits who attend the same venue are not more likely to recruit each other. A second improvement is that the RadR method expands and updates venue-based mapping (such as those used by outreach programs) and potential sampling frames (such as those used in TLS surveys, conventional cluster sampling, and census mapping). Additional venues previously unknown to the research team and not included in the original mapping exercise can be identified. Taken together, these first 2 advancements inform more targeted and efficient mapping, outreach efforts, and venue-based sampling frames. Third, the RadR method further advances the PSE field by leveraging information collected in the RDS survey to account for double counting. The original RTM makes the strong assumption that people exclusively “belong” to 1 venue. This assumption can be evaluated using the venue inquiry questions. If participants attend multiple venues, as was the case in Namibia, this information is collected and used to adjust the size estimate appropriately. Finally, the RadR method advances the PSE field and improves upon the original RTM by accounting for the proportion of the key population that is virtually *invisible* to venue-based sampling as the members in that proportion do not go to venues to find clients. The RDS methodology has often been credited with finding the more hidden members of the key population [23] and generating a more representative sample of the population [24]. The RadR method leverages this quality of the sampling design and the data collected on nonvenue attendance to calculate an inverse-probability weight for venue attendance. The inverse-probability weight adjusts the venue-based population size estimate to also account for the segment of the population that cannot be found at physical venues (eg, those who find clients or sex partners through social networking websites).

### Limitations

The RadR method assumes that the RDS sample is representative of the target population. This assumption is especially necessary for C1, the standardization parameter. If the RDS sample is representative of the target population, then R*/RDS should reflect the same relationship in the broader target population, that is, the number of people visiting a mapped venue divided by the total number of people in the population. If the representativeness of the RDS sample is of concern, several diagnostic approaches such as bottleneck plots and convergence plots are recommended to evaluate the sample [16]. These diagnostic approaches are available in RDS-A. Nonetheless, RDS has noted limitations in implementation and underlying assumptions that are difficult to prove [11,12,25], and therefore it remains uncertain if it consistently produces a truly representative sample.

We assume that study participants are not more likely to recruit other participants who attend the same venues. Although we were unable to investigate homophily (the likelihood that respondents preferentially recruit others who are similar to themselves on specified characteristics) for specific venues, we did assess this measure for overall venue attendance. Using RDS-A’s recruitment homophily function, we explored homophily by attending any venue and attending a mapped venue. In Windhoek, we found evidence for recruitment homophily with respect to reporting a mapped venue (Chi-squared *P*=.005). In Katima Mulilo, we found evidence for recruitment homophily with respect to reporting any venue attendance (Chi-squared *P*=.002) and reporting a mapped venue (Chi-squared *P*<.001). Considering how this might affect the RadR estimates, recruitment homophily would suggest that the RDS sample may not be representative of the underlying target population. This may violate our assumption that n/N=R*/RDS and impact our estimation of the components for the correction factors. One potential solution may be to use the RDS-I estimator, which is designed to account for patterns of recruitment among subgroups [25-27], and re-estimate *p*, *s*, and *t*, which are used in the simplified RadR formula. Using the RDS-I estimator, we re-estimated the Windhoek FSW population to be 1720 (95% SI: 1198-2640)—compared with the original RadR estimate of 1552 (95% SI: 1101-2,387); we re-estimated the Katima Mulilo FSW population to be 405 (95% SI: 303-574)—compared with the original RadR estimate of 453 (95% SI: 336-656). In this case, our approach to account for recruitment homophily did not result in substantially different population size estimates. However, additional research may be warranted to investigate the impact of recruitment homophily on RadR estimates in other populations.

To reduce survey fatigue, we limited our venue-inquiry questions to, at most, 3 venues. It is possible that FSWs attended more than 3 venues to find clients. Allowing for the inclusion of additional venues could expand the census mapping but may have unpredictable results for the RadR estimate, depending on whether the additional venues mentioned were mapped. Statistical modeling studies may be appropriate to determine the optimal number of venues inquired about to balance the rewards of additional information with potentially diminishing statistical returns. Investigators may also consider asking participants for the total number of venues visited before inquiring further about the 3 most often visited venues. This additional information could provide better insight into the mobility of the key population among local venues.

Although the RadR method can easily be integrated into the RDS survey with the addition of a few questions, the initial data setup can be labor intensive. Responses to the venue inquiry questions are open-ended, requiring researchers to identify and assess multiple ways of spelling the same venue name and recode these multiple references as the same venue. Researchers must then be cautious of mismatched venue names, that is, different venue names referring to the same venue or similar venue names actually referring to different venues. The potential for mismatched venue names is a limitation in this study. Future studies that implement the RadR method should collaborate with local researchers to confirm the correct matching of venue names. However, following this initial time investment, the PSE calculation is straightforward, and the data can be tabulated to validate (and update) the venue-based sampling frame and census mapping.

### Conclusions

Despite these limitations, we found that the RadR method is easily integrated into RDS studies, leveraging already collected data from a census mapping of venues during the formative assessment stage. In fact, this approach to size estimation could still be used if the census mapping and enumeration took place independently of the formative assessment for the RDS study (eg, client mapping by key population programs). Investigators must consider whether the population enumerated during the separate census mapping is the same population that is being surveyed for the RDS study. Our census mapping took place 3 weeks before the RDS study. Investigators must consider the mobility of the population when determining whether separate census mapping and enumeration can reasonably serve as a *first visit* for the target population before applying the RDS adjustment. If the target population is highly mobile, in the sense that a substantial proportion of the population either left the study site or changed venue attendance behavior in the period between the census mapping and the RDS study, then the approach detailed in this paper would not be appropriate. The RadR method improves upon the unadjusted RTM by further collecting information on multiple venues visited and the proportion of the members of the population who do not visit venues to find clients or sex partners. In addition to calculating plausible size estimates, as demonstrated here, the RadR method directly informs public health (prevention) programming by updating the census mapping and identifying venues where outreach services can take place.

## Abbreviations

CDC: Centers for Disease Control and Prevention
FSW: female sex worker
IBBSS: integrated biobehavioral surveillance study
MoHSS: Ministry of Health and Social Services
NGO: nongovernmental organization
PLWH: people living with HIV
PSE: population size estimation
RadR: respondent-driven sampling adjusted reverse tracking method
RDS: respondent-driven sampling
RTM: reverse tracking method
SI: simulation interval
TLS: time location sampling
UCSF: University of California, San Francisco

## References

[1] Schwartländer B, Stover J, Hallett T, Atun R, Avila C, Gouws E, Bartos M, Ghys PD, Opuni M, Barr D, Alsallaq R, Bollinger L, de Freitas M, Garnett G, Holmes C, Legins K, Pillay Y, Stanciole AE, McClure C, Hirnschall G, Laga M, Padian N, Investment Framework Study Group (2011). Towards an improved investment approach for an effective response to HIV/AIDS. Lancet.

[2] The Office of the U.S. Global AIDS Coordinator (2014). The United States President's Emergency Plan for AIDS Relief.

[3] Githuka G, Hladik W, Mwalili S, Cherutich P, Muthui M, Gitonga J, Maina WK, Kim AA, KAIS Study Group (2014). Populations at increased risk for HIV infection in Kenya: results from a national population-based household survey, 2012. J Acquir Immune Defic Syndr.

[4] Mumtaz G, Riedner G, Abu-Raddad LJ (2014). The emerging face of the HIV epidemic in the Middle East and North Africa. Curr Opin HIV AIDS.

[5] Okal J, Geibel S, Muraguri N, Musyoki H, Tun W, Broz D, Kuria D, Kim A, Oluoch T, Raymond HF (2013). Estimates of the size of key populations at risk for HIV infection: men who have sex with men, female sex workers and injecting drug users in Nairobi, Kenya. Sex Transm Infect.

[6] Wesson P, Reingold A, McFarland W (2017). Theoretical and empirical comparisons of methods to estimate the size of hard-to-reach populations: a systematic review. AIDS Behav.

[7] Abdul-Quader AS, Baughman AL, Hladik W (2014). Estimating the size of key populations: current status and future possibilities. Curr Opin HIV AIDS.

[8] Joint United Nations Programme on HIV/AIDS (2014). Joint United Nations Programme on HIV/AIDS.

[9] UNAIDS/WHO Working Group on Global HIV/AIDS and STI Surveillance (2010). UNAIDS.

[10] Vadivoo S, Gupte M, Adhikary R, Kohli A, Kangusamy B, Joshua V, Mathai AK, Kumar K, Mainkar M, Goswami P, IBBA Study Team (2008). Appropriateness and execution challenges of three formal size estimation methods for high-risk populations in India. AIDS.

[11] Magnani R, Sabin K, Saidel T, Heckathorn D (2005). Review of sampling hard-to-reach and hidden populations for HIV surveillance. AIDS.

[12] Johnston L, Hakim A, Dittrich S, Burnett J, Kim E, White RG (2016). A systematic review of published respondent-driven sampling surveys collecting behavioral and biologic data. AIDS Behav.

[13] Ministry of Health and Social Services (2015). Ministry of Health and Social Services.

[14] Heckathorn D (1997). Respondent-driven sampling: a new approach to the study of hidden populations. Soc Probl.

[15] Heckathorn D (2002). Respondent-driven sampling II: deriving valid population estimates from chain-referral samples of hidden populations. Soc Probl.

[16] Gile K, Johnston L, Salganik M (2015). Diagnostics for Respondent-driven Sampling. J R Stat Soc Ser A Stat Soc.

[17] Joseph L, Gyorkos T, Coupal L (1995). Bayesian estimation of disease prevalence and the parameters of diagnostic tests in the absence of a gold standard. Am J Epidemiol.

[18] Khalid FJ, Hamad FM, Othman AA, Khatib AM, Mohamed S, Ali AK, Dahoma MJ (2014). Estimating the number of people who inject drugs, female sex workers, and men who have sex with men, Unguja Island, Zanzibar: results and synthesis of multiple methods. AIDS Behav.

[19] Handcock MS, Fellows IE, Gile KJ (2014). Hard-to-Reach Population Methods Research Group.

[20] Volz E, Heckathorn D (2008). Probability based estimation theory for respondent driven sampling. J Off Stat.

[21] Johnston L, McLaughlin K, El Rhilani H, Latifi A, Toufik A, Bennani A, Alami K, Elomari B, Handcock MS (2015). Estimating the Size of hidden populations using respondent-driven sampling data: case examples from Morocco. Epidemiology.

[22] R Core Team (2014). The R Project for Statistical Computing.

[23] Kendall C, Kerr L, Gondim R, Werneck GL, Macena RH, Pontes MK, Johnston LG, Sabin K, McFarland W (2008). An empirical comparison of respondent-driven sampling, time location sampling, and snowball sampling for behavioral surveillance in men who have sex with men, Fortaleza, Brazil. AIDS Behav.

[24] McCreesh N, Frost SD, Seeley J, Katongole J, Tarsh MN, Ndunguse R, Jichi F, Lunel NL, Maher D, Johnston LG, Sonnenberg P, Copas AJ, Hayes RJ, White RG (2012). Evaluation of respondent-driven sampling. Epidemiology.

[25] Gile K, Handcock M (2010). Respondent-driven sampling: an assessment of current methodology. Sociol Methodol.

[26] Wirtz A, Mehta S, Latkin C, Zelaya CE, Galai N, Peryshkina A, Mogilnyi V, Dzhigun P, Kostetskaya I, Beyrer C (2016). Comparison of respondent driven sampling estimators to determine HIV prevalence and population characteristics among men who have sex with men in Moscow, Russia. PLoS One.

[27] Salganik M, Heckathorn D (2016). Sampling and estimation in hidden populations using respondent-driven sampling. Sociol Methodol.

